# The role of mesenchymal stem cells in cancer development

**DOI:** 10.3389/fgene.2013.00261

**Published:** 2013-11-27

**Authors:** Hiroshi Yagi, Yuko Kitagawa

**Affiliations:** Department of Surgery, School of Medicine, Keio UniversityTokyo, Japan

**Keywords:** stem cell transplantation, epithelial mesenchymal transition, cancer therapy, cytokines, stem cell niche

## Abstract

The role of mesenchymal stem cells (MSCs) in cancer development is still controversial. MSCs may promote tumor progression through immune modulation, but other tumor suppressive effects of MSCs have also beendescribed. The discrepancy between these results may arise from issues related to different tissue sources, individual donor variability, and injection timing of MSCs. The expression of critical receptors such as Toll-like receptor is variable a teach time point of treatment, which may also determine the effects of MSCs on tumor progression. However, factors released from malignant cells, as well as surrounding tissues and the vasculature, are still regarded as a “black box. ” Thus, it is still difficult to clarify the specific role of MSCs in cancer development. Whether MSCs support or suppress tumor progression is currently unclear, but it is clear that systemically administered MSCs can be recruited and migrate toward tumors. These findings are important because they can be used as a basis for initiating studies to explore the incorporation of engineered MSCs as novel anti-tumor carriers, for the development of tumor-targeted therapies.

## INTRODUCTION

Mesenchymal stem cells (MSCs) belong to a category of clinically relevant cell types that have the potential to be utilized for cell-based therapies, because complicated culturing or handling techniques are not required to yield clinically practical quantities. Traditionally, MSCs can be induced to differentiate into mesenchymal lineages such as osteoblasts, adipocytes, chondrocytes and potentially other skeletal tissue cells by culturing MSCs under defined mechanochemical conditions (Pittenger et al., 1999). MSCs are characterized by the expression of cell surface markers such as CD73, CD90, and CD105, and the absence of expression of hematopoietic lineage markers (Lama et al., 2007). Recently, there has been heightened interest into the homing and migration capacity of MSCs into tumors. Since the process of tumor progression is closely related to inflammation, the role of MSCs in carcinogenesis has emerged as an attractive new concept in cancer therapy. Although ample experimental evidence exists in support of the therapeutic potential of MSCs targeting different tumors, e.g., hepatocellular carcinoma (Gao et al., 2010), brain tumors (Nakamizo et al., 2005) and sarcoma (Khakoo et al., 2006) in different animal models, the mechanisms guiding the homing and recruitment of MSCs into tumors and their potential role in malignant tissue progression are not well understood. MSCs have been shown to promote tumor progression through immune modulation (Karnoub et al., 2007). In contrast, other studies have reported that MSCs have a suppressive effect on tumor development; e.g., via modification of Akt signaling (Khakoo et al., 2006). The use of different tissue sources, individual donor variability, and injection timing of MSCs in each experiment may have an impact on this discrepancy. The expression of critical receptors such as Toll-like receptor (TLR) is variable at various time points during the treatment (Liotta et al., 2008), which may also influence the effects of MSCs on tumor progression. Whether MSCs support or suppress tumor progression, it is clear that systemically administered MSCs can be recruited by, and migrate toward, tumors (Studeny et al., 2002; Loebinger et al., 2009). These findings are important because they can be used as a basis for studies to explore the utilization of engineered MSCs as novel carriers for delivery of anti-tumor agents to cancerous tissue, guiding the development of tumor targeted therapies.

Previous reports (Curtin et al., 2009; Tang et al., 2010) have led to a great deal of attention into the role and function of MSCs in tumors. The tropism of MSCs for tumors raised wide interest regarding their potential as a delivery vehicle for anti-cancer agents. Indeed, several reports have described the feasibility of using these cells as anti-cancer delivery vehicles because they secrete various anti-cancer molecules such as tissue necrosis factor (TNF), TNF related apoptosis inducing ligand (TRAIL), and interferon (IFN)-β by transfection. These studies demonstrated a sufficient effect in suppressing tumor progression (Nakamizo et al., 2005; Loebinger et al., 2009; Grisendi et al., 2010). However, Karnoub et al. (2007) suggested that MSCs have a supportive effect on tumor progression showing that co-injection of MSCs with cells from a breast cancer cell-line led to a higher degree of metastasis, but this effect was not significant in local tumor growth. This supportive effect on tumor growth by MSCs has also been reported in different cancers such as colon cancer, lymphoma, and melanomas (Djouad et al., 2003; Ame-Thomas et al., 2007; Shinagawa et al., 2010). In contrast, several reports showed that MSCs may have a suppressive role in tumor development via p38 mitogen-activated protein kinase (MAPK; Tian et al., 2010) or by cell fusion (Wang et al., 2012). Also, different types of tumors such as liver cancer, breast cancer, leukemia, and pancreatic cancer have been used to show a tumor suppressive effect of MSCs (Qiao et al., 2008a,b; Cousin et al., 2009; Zhu et al., 2009). On the other hand, Torsvik et al. (2010) suggested that cross-contamination of MSCs with tumor cells can enhance tumor supportive behavior. Interestingly, Klopp et al. (2011) suggested that the increased tumor mass observed in these reports can be related to increased proliferation of MSCs in the tumor.

In this review, we discuss recent findings related to mechanisms of MSC migration toward tumors, including cytokines, chemokines, as well as surrounding conditions, and, more importantly, we will also discuss the potential role of MSCs in malignant tissue progression.

## THE MESENCHYMAL STEM CELL NICHE *IN VIVO*

It was previously revealed that the primary MSC niche is in bone marrow; however, there are several reports that also identify additional peripheral locations, such as adipose tissue, salivary glands, tendon, periodontal ligament, skin, muscle, lung and, most recently, Powell et al. (2011) reported that the intestinal lamina propria is also a niche. A report by da Silva Meirelles et al. (2008) posits that an important MSC niche is the perivascular region, which, as a residual aspect of embryogenesis, might explain the presence of MSCs in many different tissue types. Stappenbeck and Miyoshi (2009) have suggested that MSCs might originate in the bone-marrow and, subsequently, be recruited distally to specific sites of tissue injury). Nonetheless, there appear to be two origins of MSC populations. One population present in peripheral locations where they interact with perivascular cells (an embryonic remnant), and a second population originating in the bone marrow, where MSCs form their primary stem cell niche and respond appropriately following tissue injury. MSCs secrete various families of active molecules, including cytokines, chemokines, and growth factors, which regulate the local bone marrow environment and modulate systemic immune responses to inflammatory events. Although there have been numerous reports demonstrating that MSCs can repair tissue by directly differentiating toward mesenchymal lineages, recent work has established that instead of, or perhaps in addition to differentiation, MSCs can enhance the differentiation of other progenitor cells into functional somatic cells. In addition, they may contribute to other aspects of local tissue repair via paracrine mechanisms.

Mesenchymal stem cells can function as immune suppressive and anti-inflammatory agents, as well as stimulators of tissue repair and regeneration. However, the difference between MSCs that are recruited from the bone marrow versus peripherally located MSCs in executing these distinct roles is unclear. Recently, Brandau et al. (2010) compared the differentiation potentials of local resident and bone marrow-derived MSCs, and suggested that the two populations were not identical. Indeed, da Silva Meirelles et al. (2008) demonstrated differences in the degree of differentiation among MSCs originating from different tissues. The homing capacity toward cancer tissues has been evaluated mostly in MSCs derived from bone marrow (Hung et al., 2005; Nakamizo et al., 2005; Loebinger et al., 2009) and less from other tissues such as adipocytes (Grisendi et al., 2010) or umbilical cord blood (Hu et al., 2011). However, once incorporated into a tumor, MSCs might contribute with other cells such as myofibroblasts, endothelial cells, pericytes, and inflammatory cells to create a microenvironment that mirror the environment of a chronic wound (Dvorak, 1986; Bergfeld and DeClerck, 2010). In this context, local tissue derived MSCs, such as from pericytes, might contribute to tumor progression.

## THE IMPACT OF ROLE DISCREPANCY ON MESENCHYMAL STEM CELLS IN CANCER DEVELOPMENT

Resident MSCs may have a critical role in maintaining the homeostasis of injured tissue through immune modulatory effects or angiogenic stimulation by secreting bioactive molecules (Lazennec and Jorgensen, 2008; Uccelli et al., 2008). Since the actual population of resident MSCs is thought to be very low and decreases with age (Caplan, 2007), the behavior of large quantities of experimentally administered MSCs is likely to be quantitatively different from the behavior of the small amount of resident MSCs. Therefore, the role of administered MSCs in cancer development is still controversial. There are various reports that describe the ability of MSCs to promote tumor progression by enhancing metastatic potential (Karnoub et al., 2007) as well as epithelial mesenchymal transition (EMT; Kabashima-Niibe et al., 2013). In contrast, Ho et al. (2013) reported tumor suppressive effects of MSCs via modification of Akt signaling, shown by the coadministration of MSCs and glioma cells resulting in a significant reduction in tumor volume and vascular density. Also, several reports have demonstrated a suppressive effect of MSCs on different types of tumors (Otsu et al., 2009; Dasari et al., 2010). These conflicting results may be due to variable experimental factors such as differences in cell source like bone marrow or fat tissue, different time points, method of cell administration, and timing. In addition, although the composition of culture media is similar to fluids present *in vivo*, it does not supply all of the bioactive factors present in the stem cell niche (Watt and Hogan, 2000). Therefore, cultured MSCs should not be considered equivalent to MSCs under physiological conditions *in vivo*. Interestingly, resident MSCs, derived from either bone marrow or local tissues, have been reported to partly contribute to the origin of cancer-associated fibroblast (CAFs) or tumor-associated myofibroblasts (Quante et al., 2011). Using a mouse model of infiammation-induced gastric cancer, Quante et al. (2011) showed that at least 20% of CAFs originate in bone marrow and are derived from MSCs. This study suggested that the number of MSCs could increase in response to cancer development and promote the malignant potential.

Whether MSCs support or suppress tumor progression, it is clear that systemically administered MSCs can be recruited and migrate toward tumors (Koc et al., 2000; Nakamizo et al., 2005). Although the effect on the tumor might be enhanced by timing or the number of administered cells, these findings can be used for the development of tumor targeted therapies by providing a basis for conducting studies to explore the incorporation of engineered MSCs as novel anti-tumor carriers (Reagan and Kaplan, 2011).

## HOMING MECHANISM OF MSCs TOWARD CANCER

Recently, interest into our understanding of MSC homing and migration into tumors has grown, and several investigators have begun to compare these two processes. Since the process of tumor progression is highly related to inflammation and, as reported by Kalluri and Weinberg (2009) that EMT is critical in cancer development, the role of MSCs in carcinogenesis is an attractive new concept in cancer therapy. In this section, we review recent reports demonstrating the homing mechanisms of MSCs. Several different mediators have been reported to be involved in this process. Some of these molecules are growth factors, chemokines and cytokines, which can regulate cell migration toward inflammatory sites. These include SDF-1 and SCF-1, CCL5/CCR5, CCR2, TNF-α, and other peptides.

### GROWTH FACTORS

Vascular endothelial growth factor (VEGF) seems to be one of the most important factors that enhances and directs stem cell motility. Indeed MSCs demonstrate intensive migratory and invasive behavior in the presence of gliomas, which express high levels of VEGF. It was reported by Ritter et al. (2008) that VEGF, as well as bFGF secreted by breast cancer cells, induced the migration of MSCs. They also demonstrated that receptors for these molecules were expressed on MSCs and that depletion of these growth factors using antibodies reduced MSC migration capacity. In addition, MSCs have been shown to migrate toward endothelial cell-derived capillaries and inhibit tumor angiogenesis (Otsu et al., 2009). Although VEGF might contribute to this phenomenon, MSCs have been shown to have opposing effects on tumors. MSCs are known to express EGF and PDGF receptors on their surface, and antibodies that block PDGF or EGF can attenuate the migration of MSCs. Those reports demonstrated that certain malignant tumors such as in glioma and breast cancer, which have highly specialized vasculature and stroma, can provide permissive environments for the selective engraftment of MSCs. Taken together, the tropism of MSCs for tumors may be mediated, at least in part, by specific growth factors and receptors expressed by MSCs, thereby using a recruitment mechanism similar to what is used in inflamed or injured tissue.

### CHEMOKINES AND CYTOKINES

Stromal cell derived factor 1α (SDF-1α), which is a well-established chemo-attractant for leukocytes, acts directly on cancer cells by stimulating proliferation through the SDF-1α receptor chemokine (C-X-C motif) receptor 4 (CXCR4) expressed on cancer cells. However, SDF-1α secretion also leads to recruitment of endothelial progenitor cells to the growing tumor, thereby promoting angiogenesis. Some reports have demonstrated that SDF-1α secreted from cancer cells enhanced MSC tropism, and an antibody that blocks SDF-1α attenuated the migration of MSCs. Infection-related cancer development processes, such as helicobacter infection in gastric cancer, can give rise to an environment conducive to the recruitment of MSCs, and this can be regulated by SDF-1 and SCF-1. It was also reported that in response to chronic helicobacter infection, bone marrow–derived cells could home to and repopulate the gastric mucosa and contribute over time to metaplasia, dysplasia, and cancer (Stoicov et al., 2013).

Chemokine (C-C motif) ligand 5 (CCL5) is one of the chemokines that can enhance stem cell migration during inflammation. It was reported by Karnoub et al. (2007) that actions of CCL5 were responsible for MSC-induced metastasis in breast cancer cells. Breast cancer cells stimulated *de novo* secretion of the chemokine CCL5 (also called RANTES) from MSC, which then acted in a paracrine fashion on cancer cells to enhance their motility, invasion and metastasis (Karnoub et al., 2007). This result shows the critical importance of CCL5–CCR5 paracrine interactions in enabling MSCs to induce cancer metastasis.

### MSC RESPONSE TO IRRADIATION AND HYPOXIA

It was reported by Klopp et al. (2007) that the role of inflammation-related cytokines and chemokines in radiation-enhanced MSC migration is significant. They identified cytokines and chemokines involved in chemotaxis towards irradiated tumor microenvironments. Low-dose irradiation of murine tumors enhanced the tropism for, and engraftment of, MSCs in irradiated tumor environments. They demonstrated that tumor cells were able to increase the production and secretion of cytokines following irradiation, e.g., VEGF, PDGF, and TGF-β, that enhanced the migratory properties of MSCs (Klopp et al., 2007). In addition, the chemokine receptor CCR2 was found to be up-regulated in MSCs exposed to irradiated tumor cells. CCR2 was undetectable or expressed at low levels in untreated cells but could be up-regulated by inflammatory cytokines such as TNF-α. In addition, inhibition of CCR2 led to a marked decrease of MSC migration *in vitro* (Ren et al., 2012). Taken together, these experiments suggest that radiation can increase the expression of inflammatory mediators that can secondarily enhance the recruitment of MSCs into the tumor microenvironment.

Hypoxia also plays a major role in tumor progression, metastasis, and poor clinical outcomes. However, the role of hypoxia in MSC recruitment to the tumor microenvironment has not been sufficiently described. It was reported by Rattigan et al. (2010) that hypoxic breast cancer cells enhance their production of IL-6, which promotes the recruitment of MSCs. Secreted IL-6 acts in a paracrine fashion on MSCs, stimulating the activation of both Stat3 and MAPK signaling pathways to enhance migratory potential and cell survival. Specifically, increased cellular migration is dependent on IL-6 signaling through the IL-6 receptor (Rattigan et al., 2010). IL-6 is generally thought of as a multifunctional cytokine that plays a role in apoptosis, cell proliferation and survival. It binds to its cognate receptor, leading to activation of the JAK/STAT signal transduction pathway. A similar pathway may contribute to the migratory capacity of MSCs toward hypoxic tumors.

### OTHER EXOGENOUS MOLECULES

Previous studies have shown that leucine, leucine-37 (LL-37), the C-terminal peptide of human cationic antimicrobial protein 18, stimulates the migration of various cell types and has similar expression patterns in tumors, damaged tissue, and in inflammatory tissue, where MSCs are prominent. These peptides also have the ability to stimulate chemotaxis of various cell types. It was reported by Coffelt et al. (2009) that LL-37 promotes ovarian tumor progression through recruitment and engraftment of MSCs into tumors, where these cells provide pro-angiogenic and immunomodulatory factors to support tumor growth and progression. Indeed, neutralization of LL-37 *in vivo* significantly reduces the engraftment of MSCs into ovarian tumor xenografts, resulting in inhibition of tumor growth, as well as disruption of the fibrovascular network. LL-37-mediated migration of MSCs into tumors likely occurs through formyl peptide receptor like-1 (Coffelt et al., 2009). These data indicate that LL-37 facilitates tumor progression through recruitment of progenitor cell populations to serve as pro-angiogenic factor-expressing tumor stromal cells. Expressed factors include IL-1 receptor antagonist, IL-6, IL-10, CCL5, VEGF, and matrix metalloproteinase-2 (MMP-2). The overall consequence of LL-37’s actions, through its recruitment of MSCs, is advancement of tumor progression.

Urokinase plasminogen activator (uPA) and urokinase plasminogen activator receptor (uPAR) are up-regulated in tumors of various origins, where they play critical roles in the development of invasive and chemo-resistant cancer phenotypes. The activation of uPA and uPAR in malignant solid tumors (brain, lung, prostate, and breast) augments MSC tropism. It was reported by Gutova et al. (2008) that chemo-attraction of MSCs to cancer cells is strongly correlated with uPAR expression levels in tumor cells, which may be important for the development of optimal stem cell-based therapies.

## DEVELOPMENT OF MSCs AS A STEM CELL THERAPY

Since it was reported by Tolar et al. (2007) that sarcoma developed following transplantation of MSCs into animals, determination of their therapeutic efficacy and safety is now required for clinical applications. From a practical perspective, MSCs seem to be a very promising cell source for use in stem cell therapies for tissue impairment, given that MSCs can home to inflamed or injured tissues, as well as tumors, likely without differentiating into somatic cells. It is important to identify the utility of MSCs in clinical settings, in the context of an understanding of their complicated mechanisms as immune and inflammatory regulators. As discussed in this chapter, the most promising clinical aspects of MSCs might be immune-modulatory and anti-inflammatory effects. However, major challenges remain in our understanding of both the actual benefits, as well as the side effects of these cells in human disease.

This review discussed key modulators regarding the importance of the migration capacity of MSCs. Controlling the level of these key factors in target tissues may be a way to increase the specificity of MSC applications in these tissues, which may also lead to a reduction in the total cell number needed for the therapy, and, in concert, may reduce potential side effects, such as malignant transformation. Receptors for the reviewed key factors expressed on MSCs, including TLR and CXCR4, can also be potentially modified genetically via transfection, which may augment the efficacy of MSCs in clinical settings and decrease the migration of MSCs to non-targeted sites.

However, the clinical application of MSCs for cancer treatment is still challenging. This review described the migratory potential of MSCs to malignant tissues, which is largely similar to MSC migration into inflammatory tissue. However, factors released from malignant cells, as well as surrounding tissues and the vasculature, are still regarded as a “black box.” Thus, remains difficult to provide a specific role for MSCs in cancer development after they migrate and home into different tissues. Although some reports have demonstrated a tumor suppressive effect of MSCs, others described a tumor supportive potential. In any case, these reports encourage the notion that MSCs may play a critical role in cancer development and may be useful as a novel therapeutic delivery system that can target malignant tumors, potentially superior to existing therapeutic molecular therapies. While MSCs can react to surrounding microenvironments, molecular therapies cannot. Thus, it is imperative that scientists continue to investigate the roles and mechanisms of MSCs in tumor progression in order to harness the therapeutic potential of MSCs to regulate both inflammatory and metastatic diseases.

For clinical applications, the methodology of administration of MSCs is crucial to determine their efficacy, since there are several reports describing the risk of capillary embolism by MSCs after intravascular administration (Furlani et al., 2009; Tatsumi et al., 2013). Additional strategies, such as co-administration of anti-coagulant or adhesion factors (Tatsumi et al., 2013), as well as engineering approaches (Karoubi et al., 2009; Houtgraaf et al., 2013), might attenuate these risks.

## CONCLUSION

In summary, there are a variety of studies that demonstrate the potential migration of MSCs toward and into tumors in response to multiple molecules secreted from tumors as well as surrounding tissues. These findings will help to clarify the role of MSCs in tumors, as well as the key mechanisms that determine whether MSCs are suppressive or supportive for tumor growth. Since MSCs are relatively easy to obtain and grow in the laboratory, we anticipate that this review may help to develop new approaches in cell therapy using MSCs to control cancer development.

## Conflict of Interest Statement

The authors declare that the research was conducted in the absence of any commercial or financial relationships that could be construed as a potential conflict of interest.
